# The MERG Suite: Tools for discovering competencies and associated learning resources

**DOI:** 10.1186/1751-0473-3-7

**Published:** 2008-05-14

**Authors:** Ravi Teja Bhupatiraju, William R Hersh, Valerie Smothers, Michael Fordis, Peter S Greene

**Affiliations:** 1 Department of Medical Informatics and Clinical Epidemiology, Oregon Health & Science University, Portland, OR, USA; 2 Johns Hopkins School of Medicine and MedBiquitous, Baltimore, MD, USA; 3 Baylor College of Medicine, Houston, TX, USA

## Abstract

**Background:**

As the demands for competency-based education grow, the need for standards-based tools to allow for publishing and discovery of competency-based learning content is more pressing. This project focused on developing federated discovery services for competency-based medical e-learning content.

**Methods:**

We built a tool suite for authoring and discovery of medical e-learning metadata. The end-user usability of the tool suite was evaluated through a web-based survey.

**Results:**

The suite, implemented as an open-source system, was evaluated to identify areas for improvement.

**Conclusion:**

The MERG suite is a starting point for organizations implementing competency-based e-learning resources.

## Background

### Competencies in healthcare

Since the Accreditation Council for Graduate Medical Education (ACGME) published the Outcomes Project and the American Board of Medical Specialties (ABMS) endorsed the general or core competencies described in the ACGME project, medical educators across the continuum have sharpened their focus on development of competency-based education [1]. Despite the challenges associated with implementing competency-based education [2], many specialty societies, boards, residency directors, and associations of medical educators are defining competencies for their specialty or discipline to guide the choice of learning activities and the assessments that learners must complete.

Concurrently, many educators are developing digital repositories of learning resources. These repositories provide rich sources of information but may be difficult to find and access, and the resources within are not always clearly associated with a formal system of competency-based education.

E-learning technology offers a way to deliver education and to assess learning and knowledge application by geographically dispersed and time-constrained physicians. Just as the adoption of electronic health records and their interoperability is being enhanced via adherence to emerging standards [3], implementation of effective e-learning may be enhanced by adoption of emerging e-learning standards [4]. The health care community is only beginning to make use of standards for e-learning. The standards organization taking the lead in adapting e-learning standards to health care is MedBiquitous [5], an ANSI-accredited organization which is devoted to advancing healthcare education through technology standards that promote professional competence, collaboration, and improved patient care.

### E-learning standards

E-learning content creation can be expensive and require considerable investments of time by authors and developers. Early e-learning systems were tightly bound to the presentation, often hard coding the content into the presentation software or more recently dynamically generating learning content from databases.

Historically when Learning Management Systems (LMSs) were used to manage the delivery of e-learning, the learning activities were built on a specific LMS, and if the LMS changed, migration of content could be difficult and costly. In a world of rapidly evolving technology and advancements in learning platforms, consumers demanded the flexibility to migrate between LMSs without sacrificing investments in course development. Further learners and educators were challenged to locate and access relevant learning materials across LMSs or the Web.

The Sharable Content Object Reference Model (SCORM) emerged to address the lack of interoperability, reusability, accessibility, and durability. SCORM is a collection of standards and specifications for packaging, describing, and running e-learning content, as well as tracking user progress through content. In addition, SCORM allows for separation of content from navigation and deployment [6]. One component of the SCORM model, the IEEE Learning Object Metadata (LOM) standard, standardizes the metadata about learning objects in an XML format. Healthcare LOM is an extension of this standard to meet the requirements of healthcare education and was designed by the MedBiquitous Learning Objects Working Group [7], a collaboration of over 30 individuals from academia, government and industry.

Work has been underway in the education sector to develop specifications for competencies and competency frameworks as well. The IEEE recently approved the Reusable Competency Definition (RCD) standard [8]. This XML standard defines a data model for describing, referencing, and exchanging competency definitions, primarily for online learning. Reusable Competency Definitions provide a way to represent the key characteristics of a competency and enable interoperability among learning systems that use competency information.

## Implementation

### The MERG suite

Leveraging e-learning standards, the investigators developed and implemented a prototype search tool called the Medical Education Resources Gateway (MERG). MERG allows users to: a) search the metadata records of competencies and e-learning content, and b) traverse linkages between selected competencies and associated content.

### Overview

MERG provides a number of tools and services that make competency-linked health-related learning content easier to locate. Content creators and maintainers can easily author metadata, create public repositories to publish the metadata, and register their repositories with MERG. They can also associate metadata with competency frameworks. The suite monitors these registered repositories, harvests the metadata and competency associations as they change, creates metadata indices, analyzes the relationships and provides various search interfaces to discover and navigate the content and competencies based on the inter-relationships of these learning elements. The MERG suite, implemented on Java and Python platforms, is a portable collection of tools for content authors, system administrators, software developers and end users. All the tools are released as open source, enabling user customization.

### Architecture of MERG Suite

**Figure 1:**
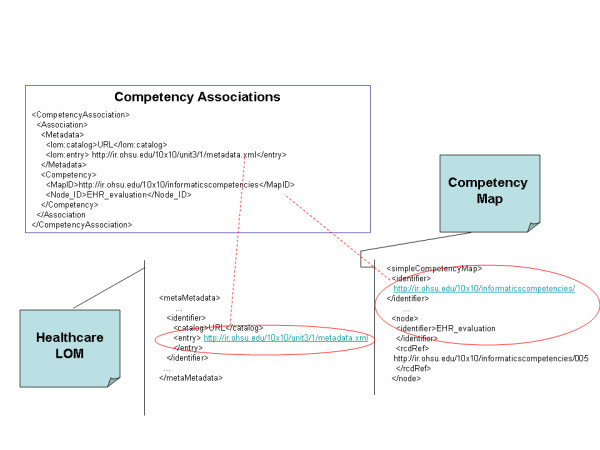
**An instance of the different metadata files participating in a content competency association (CompetencyAssociations.png).** The figure shows a learning object metadata and a competency metadata file. Each file is shown with sections containing their respective identifiers. An association metadata file is shown describing a linkage between the two identifiers.

MERG leverages the standards described in Table 1. Online learning modules use the SCORM model for interoperable learning content. The content is described using MedBiquitous Healthcare Learning Object Metadata (Healthcare LOM). Competencies are individually defined using the IEEE Reusable Competency Definition. Hierarchical relationships among the competencies are defined using the Simple Reusable Competency Map. MedBiquitous developed an XML schema to define an intermediate data structure for the specification of the associations between content and competencies. Figure 1 shows an instance of association through this schema. The information architecture of MERG is further described in previous publications [9].

**Table 1:** Standards and Specifications Used by the MERG Tool

Advanced Distributed Learning – Sharable Content Object Reference Model (ADL SCORM) [25]	A suite of standards and specifications for online education that enables interoperability of learning content across learning management systems.
MedBiquitous Healthcare Learning Object Metadata (Healthcare LOM) [26]	Extends the IEEE LOM standard and provides a format for describing healthcare learning resources including images and e-learning activities.
IEEE Reusable Competency Definition (RCD) [27]	Provides a data model for describing and exchanging competency definitions.
Simple Reusable Competency Map [28]	An IEEE proposed draft prepared by Claude Ostyn that aggregates and defines relationships among competencies.
Competency Associations [29]	A research specification developed by MedBiquitous that enables educators to create associations between content and competencies.

**Figure 2:**
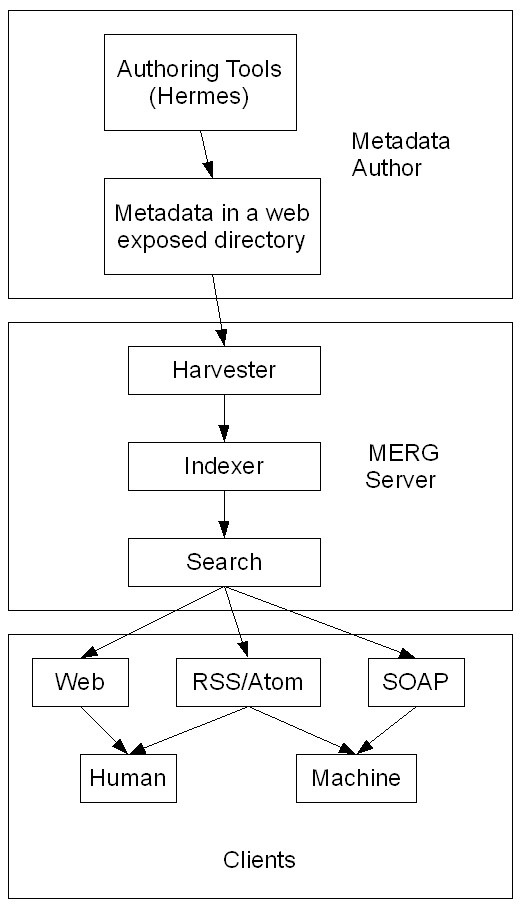
**The architecture of MERG showing the different sub-components of MERG and the interactions between them (Arch.png).** Authoring tools such has Hermes help create XML metadata to be placed in web repositories by the content owners. These are harvested periodically and indexed for the search services. Human and machine agents interface with search services via browser, web service and web feed interfaces. All communication is through XML.

Figure 2 shows the flow of information within MERG. The XML metadata created by the authoring tools is placed in a web-exposed directory along with the competency map, competency definitions, and competency associations file. These directories are periodically visited by the harvester, which supplies them to the indexer. The generated index is made available for search through web, feed and web service interfaces for both interactive and automated clients. The technical architecture is detailed here. The evolution of the suite is discussed below since the evaluation has impacted the later implementation. The earlier implementation is made available in the source package since its approach potentially has value and can be combined with the current approach once better tools become available.

### Technical choices and evolution of MERG

#### XML database approach

MERG leverages several open source products in its implementation. The original design of MERG envisioned it as an application centred on an XML database since that would have provided both storage as well as search capabilities for the metadata in its native form without the need to maintain any further transformed data representations in synchrony. We used eXist [10], an open source Java implemented XML database that provided XQuery [11] capabilities for this purpose. XQuery is a W3C standard for querying across collections of XML documents.

#### Classical text retrieval approach

We later moved away from that design since the need for emphasis on proper ranking of the search results became evident. We used traditional text search techniques on these mirrors in place of XQuery. Apache Lucene [12], an open source Java toolkit for developing search engines was used for building the search components. The results were ranked using standard TF/IDF (Term Frequency/Inverse Document Frequency) ranking. This is a commonly used approach which ranks documents by the frequency of occurrence of search terms and their uniqueness in the document collection.

Lucene allows for considerable control over search strategies in an elegant way. We currently do not have enough data for rationally arguing for alternate strategies for this metadata corpus but opportunities exist in the future for the evolution of the product. Moving to Lucene also increased the search performance, but competency-based searching has not yet been implemented. We are now using the file system as mirror storage for the repositories.

There are currently ongoing open source efforts towards providing full text search support for eXist [13] and the results might be relevant to our implementation. Once these options become mature, the project may be able merge both the search approaches for a more natural design.

#### Application Platforms

We used Karrigell [14], an open source pure Python based web server for Python based designs and used GlassFish [15], an open source Java Application Server for the later Java based designs. The early implementation was implemented in Python while the final implementation was on the Java platform with the exception of the simple harvester script, which is implemented Python invoking the GNU Wget [16] which provides the mirroring functionality.

#### Web Interface

**Figure 3:**
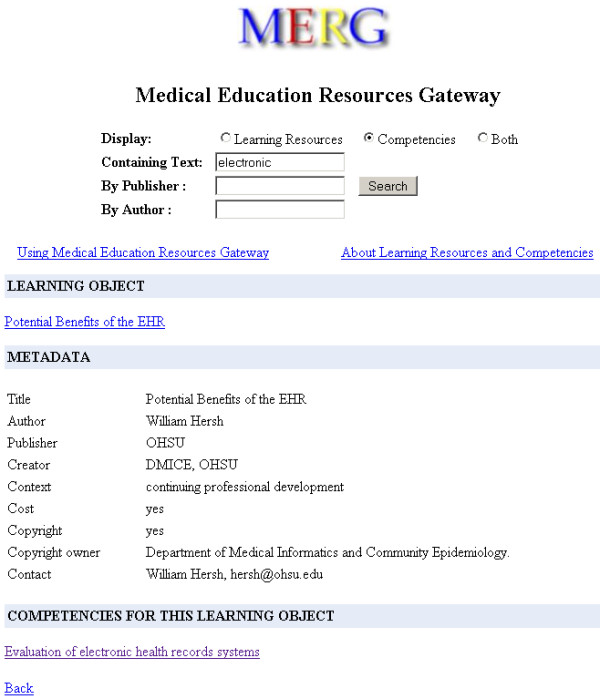
**MERG's search interface and its display of a learning object metadata record (LOM.png).** This LOM record has a single competency associated with it. MERG looks this information up from the metadata files following the MedBiquitous Competency Association schema [Table 1].

**Figure 4:**
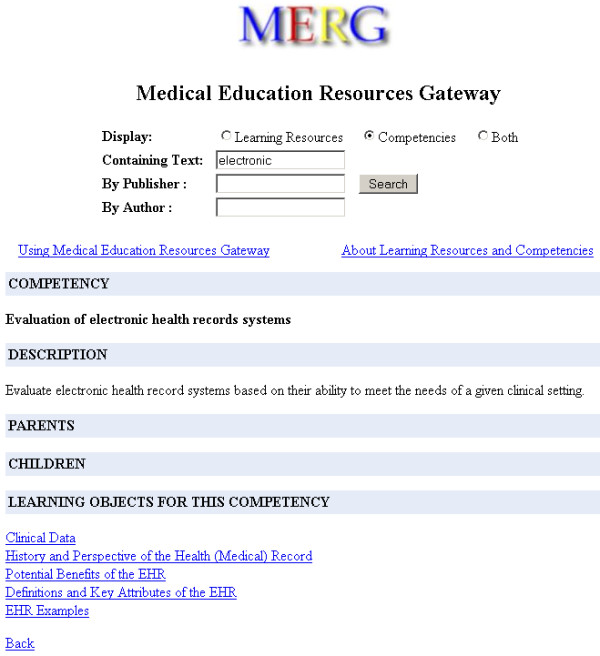
**MERG's search interface and its display of a competency record (Comp.png).** This competency record has no parent or child competencies. Other competencies may be represented in as trees drilling down to more specific and focused competencies. MERG looks this information up using the metadata files conforming to Simple RCD Map specification [Table 1]. This competency has 5 Learning Objects mapped to it. As with LOM records, MERG uses MedBiquitous Competency Association standard [Table 1] to look this information up.

**Figure 5:**
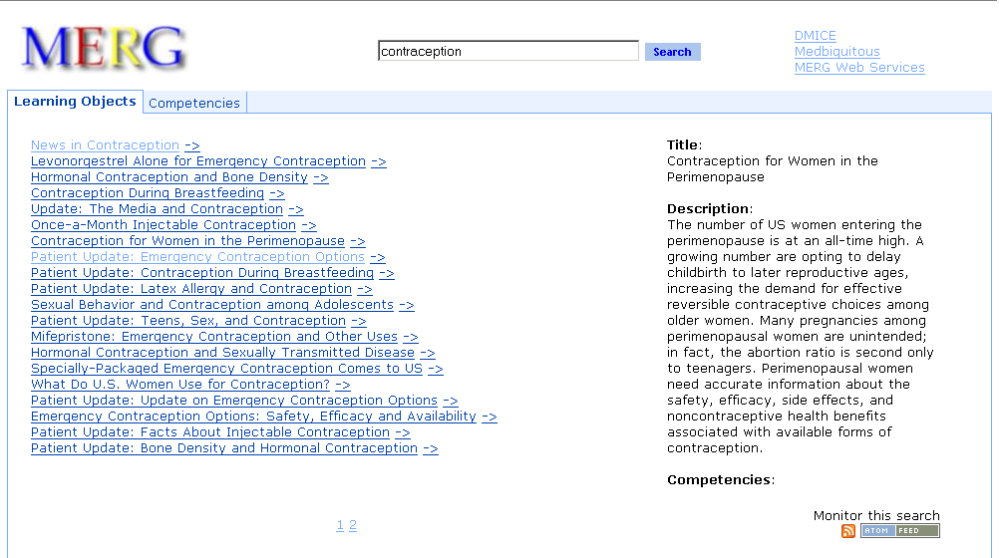
**Updated web user interface (MERGv2.png).** Updated user interface for MERG in development. The new user interface uses modern web paradigms to deliver a more responsive user interface. Cosmetic improvements were made as well.

The early web interfaces focused on the retrieval aspects of the design [Figure 3, 4]. The user feedback enabled us to improve the usability of the later design [Figure 5]. The web interface was revamped to make better use of screen real estate using a tab based interface. The LOM and Competency results as well as metadata detail screens were merged into the search page. The new interface moved to use AJAX (Asynchronous JavaScript and XML) techniques. DWR (Direct Web Remoting) [17] an open source Java library was used for AJAX functionality. Pagination features were enabled via AJAX allowing smaller result sets to be returned and updated on the same page. As a result of these changes, the new implementation became noticeably faster when compared to the previous design.

#### Feeds

The RSS and ATOM features of our application were enabled by the library provided by the ROME project [18].

#### Hermes

Hermes is a pure Java Swing application that uses DOM4J [19] for XML parsing inputs and Apache project's Velocity as the template engine [20] for generating outputs.

#### Addressing various user profiles

The audience of MERG is a diverse group. It consists of authors who create competency-based content and metadata, system administrators who create the local metadata repositories, repository maintainers who update them, content consumers who search the aggregated repositories and software developers who may build on top of the existing infrastructure. Each of these user classes have distinct technical backgrounds and perform very different tasks. Addressing these multiple perspectives is one of the challenges of MERG. The modularity of MERG allows it to direct specific sub-components of the system to the user profiles that directly interact with them. Each of the user profiles, the tasks they perform, the sub-components the users interact with and some of the design decision decisions that went in to address those are described below.

##### Authors

**Figure 6:**
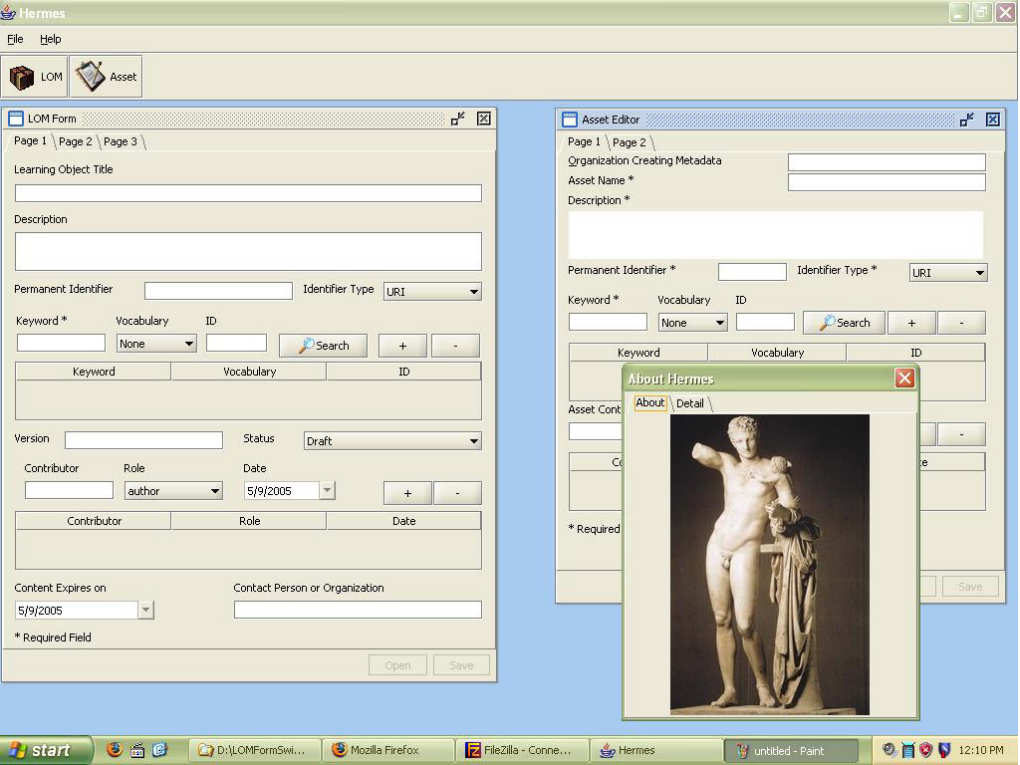
**Hermes metadata editor showing the learning object and asset metadata editing dialogs (Hermes.jpg).** Hermes provides a visual interface for editing the XML metadata files describing content. A learning object is built from one or more assets. Audio, video files, web pages etc are considered assets in SCORM. The LMS uses this metadata to orchestrate content presentation.

To support authors in creating metadata, the MERG suite includes Hermes, a metadata authoring tool for the Healthcare LOM. Hermes provides a graphical user interface (GUI) for creating and editing metadata, removing the need to manually manage the XML files through a text or a generic XML editor. MERG features a simple sample network-based look-up service to query Medical Subject Headings (MeSH) terms, which are loaded into Hermes. Healthcare LOM supports the use of MeSH Terms as structured keywords. This service feature is a sample stub and is intended to be replaced by similar but more pertinent custom look-up services to vocabulary subsets specific to the needs of organizations. Hermes provides editors for the learning object and asset metadata files. Hermes is a Java web application and can be easily deployed to most desktop environments. Figure 6 shows the user interface of the Hermes metadata editor. The LOM and Asset metadata editors are shown within the Multi Document Interface.

##### System administrators

To guide the system administrators who setup the repositories, the MERG suite provides a specification for the repository structure and an interface for registering repositories with MERG. It has a harvester component that visits registered repositories at a specified frequency to update its local copy of repository content. The repository structure and harvester functionality are intentionally kept as simple as possible. Individuals with skills to set up a Web server should have the skills to set up a metadata repository that can be harvested by MERG.

##### Repository maintainers

To simplify the task of repository maintainers, MERG repositories are specified with a simple structure intended for use by non-technical audiences, namely content and metadata authors. The repository is maintained by placing all the metadata files into a flat Web exposed directory. Metadata files conform to the Healthcare LOM standard and are assigned filenames with '.xml' extensions. The server's directory listing feature can be used to create a directory of the files if such a feature is available. Alternatively, the listing can be manually created, although this is not usually recommended since more effort or additional tools may be required to synchronize the listing with others in the repository. The metadata files need not be specially named or arranged, but are to be placed in the same directory. Competency definitions, maps, and competency-metadata associations are placed in the Web-exposed directory with the metadata files. MERG's Indexer is able to recognize the file types and processes them accordingly.

##### Software Developers

To assist the software developers in developing extensions and other value added services, MERG uses the W3C SOAP (Simple Object Access Protocol) 1.2 standard [21] to provide a remote interface to query the MERG search engine for metadata. This allows software developers to incorporate the end user functions of MERG into their applications without worrying about web user interface changes that may potentially occur in future.

##### Content consumers

Through a simple web interface, MERG enables users to query across multiple metadata repositories registered with MERG. The users navigate between related competencies and learning objects. Figure 3 shows the MERG search interface after a search has been performed and a LOM result from the search has been selected. The selected LOM record has a single competency associated with it. Figure 4 shows a selected competency record after a search. The selected competency record has no parent or child competencies.

**Figure 7:**
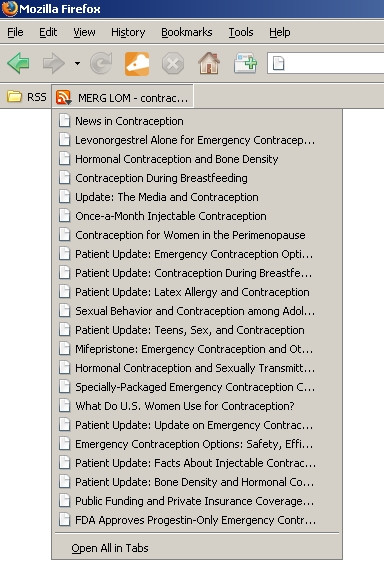
**Web feeds in MERG (Feed.jpg).** Figure showing a LOM feed for the search term "contraception" that the user dragged and dropped into in his browser's panel. When the users wish to monitor their searches for new content, they may do so with little effort using standard feed readers.

Users wishing to be alerted to new content received on the MERG repository may provide specified search criteria and monitor search results using syndication feeds. The users will be presented feed URLs for each search they perform. They may then bookmark these in their feed reader, as many feed readers support user notifications when the feed content changes. MERG supports the latest standards for both RSS and ATOM, thereby allowing a very broad range of feed readers to be used. Figure 7 shows a LOM feed for the search term "contraception" that the user dragged and dropped into the browser's (Firefox) bookmarks toolbar.

### A use case for the MERG Suite

A single coherent hypothetical use case scenario where various MERG Suite components come into play is described as follows.

Dr. X is a gastroenterologist and a medical educator who has prepared an online course for internists in his institution, Springfield Medical Center (SMC), on recent trends in hepatology. SMC uses a SCORM-compliant Learning Management System to deliver its content. The CME co-ordinator, Ms. Y, manages the various courses that either have been purchased or developed in house. Since Dr. X's content has been developed in house and the author has no special knowledge of the workings of Learning Management Systems, Ms. Y helps him by creating the metadata for his content and placing his course in the learning management system. Ms. Y herself has no special technical skills and is only modestly familiar with XML and certainly not with all the rules associated with authoring valid metadata. She uses the Hermes metadata editor to create the metadata for Dr. X's content through a friendly user interface.

A few months later, Dr. X's content has been well received. Through the word of mouth, physicians from other local institutions have requested access to his course. Dr. X and Ms. Y see potential to offer this course to the broader community. Dr. X would like to update the content from time to time as new advances are made in the subject area and Ms. Y sets him up with Hermes to edit the metadata. Ms. Y asks Mr. Z, an SMC network administrator, to set up a metadata and content repository so that SMC's courses can be searched by the broader community. She also asks Mr. Z to work with her to associate the content to a new competency-framework for gastroenterology that is being used by the residency program. Mr. Z is the web master for SMC web site. He downloads the competency map and competency definitions form a national gastroenterology association site. He finds that he simply has to place the metadata and competency files in a folder and enable directory browsing on the folder. He registers the URL of this repository with MERG search engine. MERG harvests and indexes the content and the competency associations. The content itself is placed in a password protected folder.

Dr. W, an internist from rural Pennsylvania, searches MERG for Hepatology content. He finds Dr. X's course and contacts Ms. Y at SMC. He registers for the course and Ms. Y authorizes Dr. W to view the content. Dr. W completes the course and subscribes to the RSS feed from the Hepatology search using his web browser. He will be notified as new Hepatology CME content is registered into MERG.

Ms. Y mentions MERG to her EMR provider, Z-Med. Z-Med looks into MERG and finds that it provides a web services interface to its search. Z-Med has been looking to integrate CME opportunities into its electronic health record system. Z-Med comes up with a plan to integrate links to CME content in its physician portal.

## Methods

In order to test the usability of the system, we developed metadata for two content domains:

1. The course, *Introduction to Biomedical Informatics*, was developed by one of the authors (WRH) and is taught in the biomedical informatics graduate program at Oregon Health & Science University (OHSU) and the AMIA-OHSU 10 × 10 Program [22].

2. Content from *Contraception Online *[23] was made available by another author (MF) and offers a variety of learning resources for healthcare providers and patients on a range of topics related to contraception. Resources included didactic slide presentations, interactive cases, monographs, and patient education handouts.

MERG Suite underwent internal iterative developmental evaluation towards the design of Hermes and the server side tools. The feasibility of the prototypes were tested using the above two real world metadata collections. Since the web search component of MERG was expected to service most users, we conducted a survey of the potential users of the systems. After receiving approval from the OHSU Institutional Review Board, we administered the survey using Survey Monkey [24]. Participating medical educators were recruited using several email lists (e.g., the MED-ED email list of the Association of American Medical Colleges; the DR-ED email list of Michigan State University; and the MedBiquitous Competencies, Learning Objects, and Virtual Patient Working Groups). The survey consisted of two parts. The first part focused on the use of competencies in medical education. The second part of the survey focused on experience with MERG suite and was administered after participants had an opportunity to apply the tool to one of the two content areas – medical informatics or contraceptive topics in women's health.

The survey instrument included questions about participants' educational roles and responsibilities, their use and the availability of competencies for the discipline(s) in which they teach, and their views on the value of a tool that would enable searching for e-learning content by using competency search terms. Each participant was asked to use our prototype system with its candidate competencies and content and to provide feedback to guide further development.

## Results

A total of 42 individuals took part in our survey. As seen in Tables 2, 3, 4, the participants serve in various medical education roles, with responsibilities for educating a diverse mix of learners, including physicians (the most frequently represented target audience, graduate students, and nurses and allied health professionals. With respect to physician education, over three-quarters had responsibilities in undergraduate medical education, two-thirds for graduate medical education, and slightly less than half for continuing medical education. However, all were frequently involved in developing learning materials. Nearly 80% (30/38) stated that competencies were available to them, with most citing the ACGME competencies, rather than discipline-specific competencies. As shown in Table 5, most of survey participants had used competencies recently.

**Table 2:** Professional role(s) of respondents.

Administrator	16 (42.1%)
Clinician educator	15 (39.5%)
Clinician	7 (18.4%)
Course director	11 (28.9%)
Curriculum developer	19 (50.0%)
Researcher	13 (34.2%)
Teacher or course faculty member	18 (47.4%)
Other	6 (15.8%)

(Since individuals may have more than one role, the results reflect the percentage with each role out of the total and the sum exceeds 100%.)

**Table 3:** Educational target audiences of respondents. (Total may exceed 100%.)

Medical students	30 (78.9%)
Graduate students	18 (47.4%)
Residents/fellows	25 (65.8%)
Physicians in practice	18 (47.4%)
Nurses, Physician assistants, and other allied health professionals	12 (31.6%)

**Table 4:** Frequency of respondents' development of lectures, instructional materials, syllabi, or other scholarly educational products for use in teaching.

Never	2 (6.2%)
Less than once a year	1 (3.1%)
1–2 times a year	2 (6.2%)
3–6 times a year	9 (28.1%)
Monthly	11 (34.4%)
Weekly	7 (21.9%)

**Table 5:** Most recent use of competencies to develop educational materials.

Never	5 (15.6%)
1 to 2 years ago	2 (6.2%)
6 months to 1 year ago	1 (3.1%)
3 to 6 months ago	7 (21.9%)
Within the last 3 months	17 (53.1%)

Table 6 shows their perceptions about the competencies. While over 80% described comfort with using competencies to develop educational materials, over 40% reported that significant barriers exist to using competencies for that purpose. Furthermore over 90% felt that linking learning resources to competencies would be useful and 88% would use a tool that facilitated such linkage. Finally over half believed that access to such a tool may change how they design instruction.

**Table 6:** Likert scale ratings of opinions concerning competencies.

	Strongly Agree	Agree	Neutral (do not know)	Disagree	Strongly Disagree
I am comfortable in using the available competencies to inform instructional interventions.	10 (37.0%)	12 (44.4%)	3 (11.1%)	1 (3.7%)	1 (3.7%)
Significant barriers exist to using competencies to develop education or educational products.	2 (6.2%)	12 (37.5%)	9 (28.1%)	9 (28.1%)	0 (0.0%)
I have an interest in learning more about the competencies available for my specialty or discipline.	15 (46.9%)	15 (46.9%)	2 (6.2%)	0 (0.0%)	0 (0.0%)
It would be useful to link learning resources to the competencies that those resources address.	21 (65.6%)	9 (28.1%)	2 (6.2%)	0 (0.0%)	0 (0.0%)
I would use a search tool that linked learning resources to competencies and linked competencies to relevant learning resources.	19 (59.4%)	9 (28.1%)	4 (12.5%)	0 (0.0%)	0 (0.0%)
Having access to a tool such as this may change how I go about designing instruction.	6 (18.8%)	12 (37.5%)	11 (34.4%)	3 (9.4%)	0 (0.0%)

With respect to the barriers in using competencies, respondents elaborated in the free-text comments:

1. Available competency sets do not align with curriculum goals.

2. There are inadequate numbers of competency sets.

3. The faculty does not yet understand the concept of competency-based education.

4. Available competency sets are not practical or are vague or unproven.

5. Educators have trouble choosing between competing competency sets.

6. There is difficulty in adopting competency sets within traditional curricula.

7. Creating competency-based assessment tools is difficult.

8. Competency sets add to information overload.

Although participants expressed enthusiasm for the MERG-type general search tool, their reaction to our prototype system was mixed. As shown in Table 7, nearly half of the participants indicated that the MERG search tool was easy to use. However, assessments of the usefulness of accessing learning resources were mixed as indicated by the responses to three additional questions. Over three-quarters (24/31, 77.4%) of participants reported that the search result pages did not show sufficient information, and about half (16/31,51.6%) reported that the search function was useful and a similar proportion (17/31,54.8%) reported that the links between competencies and content were useful.

**Table 7:** Usability of MERG.

	How easy or difficult was the tool to use?	How easy or difficult is it to access learning resources through MERG?
Very difficult	0 (0.0%)	4 (12.9%)
Difficult	10 (32.3%)	10 (32.3%)
Neutral	6 (19.4%)	7 (22.6%)
Easy	13 (41.9%)	8 (25.8%)
Very easy	2 (6.5%)	2 (6.5%)

Although our intent was to assess respondents use of our search system linking competencies to relevant learning objects using limited data sets for illustrative purposes, survey participants in their comments focused more on the narrowness of available learning objects rather than on any challenges encountered in the use of the search system. This may be that in our instructions we did not communicate the limited nature of learning objects available for testing and thus did not manage expectations and/or the search tool itself was intuitive for most users and the challenges encountered related more to their inability to identify a wide variety of resources. The feedback we gathered from the study guided us in updating the web user interface. Figure 5 shows the updated user interface for MERG. The current user interface is more fully featured and designed as a single page view with a tabbed interface. It is also more responsive and incorporates some cosmetic improvements.

## Conclusion

Our study engaged a group of medical educators that understood and endorsed the competency-based approach to learning; although they did indicate that the competency sets specific to their teaching are currently limited. While they agreed in principle with the approach taken through development of the MERG search tool, the participants focused on the comparatively scant content generated from their searches. In retrospect, participants should have been alerted to the narrowness of the current content knowledge-base which was assembled only for usability evaluation purposes. More specific instruction should have been provided to participants explaining our interests in testing the functionality of the search tool and factors related to ease of use, rather than investigations related to the comprehensive nature of search returns.

Notwithstanding the issue of search return volume, the results from this assessment of MERG suite functionality indicate that overall the professionals who tested it found it easy to use and were in agreement with the usefulness of the approach. With the addition of a wider range of content and linked repositories, quite different testing results would likely be realized and greater insights into functionalities of interest might be revealed.

The future utility of MERG will depend on the availability of standardized, competencies that are linked to content.

### Barriers to adoption

While the important role of competencies is now recognized in the educational community and standards for authoring competencies are available, only a small portion of content is linked to competencies. Tools such as the MERG suite may lower the barrier of entry in developing and distributing competency-linked content from a technical perspective by providing tools that shield the users from the details of the specification and by offering guidance for setting up the repositories. However, additional research is needed with respect to effective use of competencies. Experiences in domains such as U.S. Public Health Service, which appear to be at the forefront in designing and applying competencies for health professional education, may inform future development in this area.

MERG has been designed as a modular application with loose coupling between components. While this approach has distinct advantages, it does pose challenges in deployment. Although not a substantial service at present, our aim is that the MERG tool kit serve as an evolutionary platform for implementing such services in the future.

## Availability and requirements

Project name: MERG Suite

Project home page: 

Operating system(s): Windows 98 or higher, Unix-based OS.

Programming language: Java, Python, Groovy

Other requirements: Java 1.6 or higher, Python 2.5 or higher

License: GNU GPL.

Any restrictions to use by non-academics: None

## Competing interests

The authors declare that they have no competing interests.

## Authors' contributions

RTB is a doctoral candidate in the Biomedical Informatics PhD program at Oregon Health & Science University. RTB designed and programmed the systems.

WRH is Professor and Chair of the Department of Medical Informatics and Clinical Epidemiology and provided leadership as Principal Investigator of the project. He also provided part of the metadata used for testing the systems from a course taught by him as well as designed and disseminated the survey used for evaluation.

VS is the Deputy Director of MedBiquitous. VS provided the expertise for working with standards from MedBiquitous.

MF is the Senior Associate Dean for Continuing Medical Education at Baylor College of Medicine and Director for the Center for Collaborative and Interactive Technologies. MF assisted with women's health portion of the metadata content that was used to test the readiness of the system for real world metadata and contributed to the survey design

PSG is the Executive Director of MedBiquitous and provided leadership as a Co-Investigator on the project.
